# Reply to “Lobular capillary hemangioma with halo phenomenon”

**DOI:** 10.1016/j.jdcr.2025.01.004

**Published:** 2025-01-28

**Authors:** Mohammed Ibrahim AlJasser

**Affiliations:** a College of Medicine, King Saud bin Abdulaziz University for Health Sciences, Riyadh, Saudi Arabia; b King Abdullah International Medical Research Center, Riyadh, Saudi Arabia; c Division of Dermatology, Ministry of National Guard Health Affairs, Riyadh, Saudi Arabia

**Keywords:** angiokeratoma, cherry angioma, depigmentation, dermoscopy, halo nevus, halo phenomenon, hypopigmentation, postinflammatory hypopigmentation, Sutton nevus, vascular lesion, vitiligo

*To the Editor:* I would like to thank Dr Cohen et al for their interest in our report entitled “Halo angiokeratoma.” The authors highlight the possibility that postinflammatory hypopigmentation may serve as an alternative mechanism for the formation of white halos around vascular lesions. They also support that with a well-written report of white halo developing around lobular capillary hemangioma.

White macules or patches in dermatology can be caused by a wide range of pathologies. They are usually due to changes in melanin but can also be due to vascular changes (as seen in nevus anemicus). We aimed to emphasize those 2 possible origins of white color (ie melanin and hemoglobin), given that the patient in our report presented with a vascular lesion (angiokeratoma). We mentioned that white halos can be pale (vascular in origin) or depigmented (melanocytic in origin). Examples of potential vascular origin of the white halo include eruptive pseudoangiomatosis,^1^ pale halo around some cherry angiomas,^2^ and the blanched halo that can be seen around some vascular malformations.^3^

Halo nevi and vitiligo are both characterized by depigmentation. Our patient had vitiligo, which made the discussion more toward depigmentation. Hypopigmentation (rather than depigmentation) can be seen in early vitiligo. Hypochromic areas are considered one of the main signs of vitiligo activity.^4^ Melanocytes and CD8^+^ T cells were shown to be present in hypochromic areas in patients with active vitiligo.^5^

In summary, white halos around vascular lesions can be melanocytic (hypo- or depigmented) or vascular in origin.

## Conflicts of interest

None disclosed.
